# Capsaicin and Dihydrocapsaicin Determination in Chili Pepper Genotypes Using Ultra-Fast Liquid Chromatography

**DOI:** 10.3390/molecules19056474

**Published:** 2014-05-21

**Authors:** Magaji G. Usman, Mohd Y. Rafii, Mohd R. Ismail, Md. Abdul Malek, Mohammad Abdul Latif

**Affiliations:** 1 Department of Crop Science, Faculty of Agriculture, Universiti Putra Malaysia, 43400 UPM, Serdang, Selangor, Malaysia; 2 Institute of Tropical Agriculture, Universiti Putra Malaysia, 43400 UPM, Serdang, Selangor, Malaysia; 3 Bangladesh Institute of Nuclear Agriculture, Mymensingh-2202, Bangladesh; 4 Bangladesh Rice Research Institute, Gazipur-1701, Bangladesh

**Keywords:** capsaicin, dihydrocapsaicin, ultra-fast liquid chromatography, chili pepper, Scoville heat units

## Abstract

Research was carried out to estimate the levels of capsaicin and dihydrocapsaicin that may be found in some heat tolerant chili pepper genotypes and to determine the degree of pungency as well as percentage capsaicin content of each of the analyzed peppers. A sensitive, precise, and specific ultra fast liquid chromatographic (UFLC) system was used for the separation, identification and quantitation of the capsaicinoids and the extraction solvent was acetonitrile. The method validation parameters, including linearity, precision, accuracy and recovery, yielded good results. Thus, the limit of detection was 0.045 µg/kg and 0.151 µg/kg for capsaicin and dihydrocapsaicin, respectively, whereas the limit of quantitation was 0.11 µg/kg and 0.368 µg/kg for capsaicin and dihydrocapsaicin. The calibration graph was linear from 0.05 to 0.50 µg/g for UFLC analysis. The inter- and intra-day precisions (relative standard deviation) were <5.0% for capsaicin and <9.9% for dihydrocapsaicin while the average recoveries obtained were quantitative (89.4%–90.1% for capsaicin, 92.4%–95.2% for dihydrocapsaicin), indicating good accuracy of the UFLC method. AVPP0705, AVPP0506, AVPP0104, AVPP0002, C05573 and AVPP0805 showed the highest concentration of capsaicin (12,776, 5,828, 4,393, 4,760, 3,764 and 4,120 µg/kg) and the highest pungency level, whereas AVPP9703, AVPP0512, AVPP0307, AVPP0803 and AVPP0102 recorded no detection of capsaicin and hence were non-pungent. All chili peppers studied except AVPP9703, AVPP0512, AVPP0307, AVPP0803 and AVPP0102 could serve as potential sources of capsaicin. On the other hand, only genotypes AVPP0506, AVPP0104, AVPP0002, C05573 and AVPP0805 gave a % capsaicin content that falls within the pungency limit that could make them recommendable as potential sources of capsaicin for the pharmaceutical industry.

## 1. Introduction

**Figure 1:**
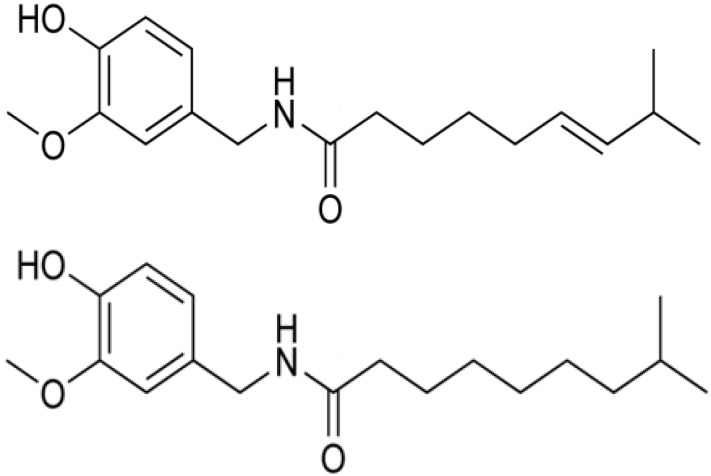
Structures of capsaicin (top) and dihydrocapsaicin (bottom).

Chili pepper, which belongs to the genus *Capsicum* contains capsaicinoids, alkaloid compounds that produce the pungency associated with eating chilies [1]. The two major capsaicinoids are capsaicin (*N-*[(4-hydroxy-3-methoxypheny) methyl]-8-methyl-*E-*6-nonenamide) and dihydrocapsaicin (*N-*[(4-hydroxy-3-methoxyphenyl)methyl]-8-methyl-6-nonanamide) which comprise over 90% of the total present in the fruit [2] (Figure 1). Capsaicin is a flavourless, odourless and colourless compound found in varying amounts in peppers. Capsaicinoids are only found in the *Capsicum* genus and are bioactive molecules currently relevant in medical and food sciences [3,4,5] as well as in the defense weapon industry [6]. Capsaicinoids occur in the placental tissue of pepper fruits [7], and their biosynthesis depends on a complex and still not fully characterized enzymatic pathway.

Capsaicin is the active element in pepper, which accounts for its prominent pharmaceutical and antioxidant properties. Research has shown that the more the capsaicin, the hotter the pepper, and the higher the antioxidant level. It is the principal pungent and irritating constituent of hot peppers that produce the pungency associated with the eating of chilies. Capsaicin and other capsaicinoids produce a number of physiological and pharmacological effects on the cardiovascular system and gastro-intestinal track [8,9,10,11,12]. Capsaicin in peppers has been shown to slightly control appetite – at least briefly. It has also been reported to raise the body temperature [9]. That warming effect may have another benefit that may help with weight loss. The temperature at which chili peppers are grown, the position of the fruit on the plant, age of the plant and light intensity are all factors affecting the total amount of capsaicin in a given chili pepper variety. Capsaicinoid levels depend on the genotype [13] and also change during fruit development [14,15,16]. Moreover, environmental and nutritional conditions occurring during the cultivation of peppers can affect the capsaicinoid content. For instance, significant differences in pungency were found in double-haploid chili plants grown in five different plots of the same field [17], and the total capsaicinoid content in “Padrón” pepper fruits developed in summer was found to be larger than in those fruits developed in autumn [18].

The large variability in capsaicinoid content found naturally in pepper genotypes is a critical point in breeding and production. For instance, capsaicin and dihydrocapsaicin contents ranged from 2 to 6,639 mg/kg in eight different pepper genotypes [19]. Therefore, there is a requirement for analytical techniques able to determine very low amounts of capsaicinoids. These techniques should also be capable of determining amounts of the different capsaicinoid molecules, which have very similar chemical structures. These requirements are met by HPLC-MS (mass spectrometry) techniques, which have a high selectivity and sensitivity and have been used for the determination of capsaicinoids in forensic, medical, and food sciences [19,20,21,22].

The first method developed for the measurement of chili pungency was the Scoville Organoleptic Test [23]. A group of five testers assess a water-diluted chili sample and then records the hot flavor level. Serial dilution of the sample is necessary to make the pungency undetectable. A number is assigned to each hot pepper according to the dilution test and expressed it as a scale called the Scoville Organoleptic Scale assigned by Pharmacist Wilbur Scoville [23]. The heat levels vary widely from 0–500,000 Scoville heat units (SHU). They are classified as:

- (0–700 SHU) non-pungent
- (700–3,000 SHU) mildly pungent
- (25, 000–70,000 SHU) highly pungent
- (3,000–25,000 SHU) moderately pungent
- (>80,000 SHU) very highly pungent [24]

However, the traditional method has been replaced by a number of instrumental methods such as Gas chromatography (GC), Gas Chromatography-Mass Spectrometry (GC-MS) and high performance liquid chromatography (HPLC) which are more reliable and accurate. Researchers need reliable, safe and standard methods that could be useful for comparing pungency levels among different samples or genotypes of chili. In this research Ultra Fast Liquid Chromatography (UFLC) was used, which is faster and simpler than conventional HPLC. The transition from LC to ultra fast LC reduces some of the limitations normally associated with LC. With HPLC, when analyzing multiple samples, each of which takes a long time to complete, the need to conduct re-analysis for whatever reason can result in product delays. However, with ultra fast liquid chromatography, an ultra high speed analysis could be achieved. This means of shortening of the time required to complete the analysis, thereby reducing the risks associated with time-sensitive analyses. This research also aims at estimating the levels of capsaicin and dihydrocapsaicin that may be found in some heat tolerant pepper varieties and to determine the degree of pungency as well as percentage of capsaicins of each of the analyzed peppers which could be used in pharmaceuticals.

## 2. Results and Discussion

### 2.1. Optimization of UFLC Separation Condition

**Figure 2:**
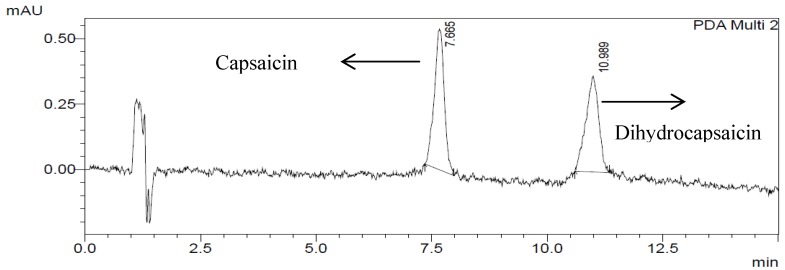
Chromatogram of capsaicin and dihydrocapsaicin (0.50 µg/g) using UV detection at 280 nm.

The chromatographic conditions used were optimized with the aim of obtaining the separation with of adjacent peaks with good resolution within a short analysis time. A binary mixture of 1% acetic acid (aq)—acetonitrile was used as described by the AOAC [25] official method. Under the optimal isocratic conditions, both capsaicin (retention time 7.665 min) and dihydrocapsaicin (retention time 10.989 min) were separated within 15 min (Figure 2). Since the molecular structures of both capsaicin and dihydrocapsaicin are very similar, the maximum absorption wavelengths determined by PDA are also nearly the same and found to be 280.8 and 279.6 nm. The PDA using Shimadzu LC solution software detects the absorbance at the chosen wavelengths of the capsaicinoids and simultaneously provides their absorption spectra. Identification of compounds was achieved by retention time and absorption spectrum of standard and sample. However, both compounds were detected with PDA at 280 nm.

### 2.2. Method Validation

The validation and verification of the UFLC method was carried out according to international guidelines for validation of the Food and Drug Administration (FDA) and National Association of Testing Authorities (NATA):

*Linearity* The linearity was found to be in the range of 0.05 to 0.50 µg/g for both compounds*.* Standard solutions were prepared from a stock solution of capsaicin and dihydrocapsaicin using six serial dilutions at 0.50, 0.40, 0.30, 0.20, 0.10 and 0.5 µg/g (acceptable by NATA). Each solution was injected three times and the average values of the triplicate analysis were presented in Table 1. The standard solutions were run on the ultra high performance liquid chromatography and the standard curves were generated by plotting peak area against concentration. The external calibration curves (Supplementary Data) were found at r^2^ = 0.9999 for capsaicin and r^2^ = 0.9996 for dihydrocapsaicin. The values of r^2^ were highly significant confirming the good linearity of the method. The regression line equations were:

Y = 18419x + 188.83 (capsaicins); Y = 15797x + 148.72 (dihydrocapsaicins)
(1)

**Table 1:** Calibration data of the UFLC method for the determination of capsaicinoids (µg/g).

Capsaicinoids	Linear Range	R^2^	Ret. Time	Average Peak Area	SD	% RSD
Capsaicin	0.05–0.50	0.9999	7.665	4947.1	27.7	0.56
Dihydrocapsaicin	0.05–0.50	0.9996	10.989	4229.6	58.1	1.37

*n* = 3.

The y-intercept means that when the concentration (x) = 0, then the peak areas of capsaicin and dihydrocapsaicin would be 189 and 149, respectively. The lowest measured values in this investigation for capsaicin and dihydrocapsaicin were 1,106 and 949 respectively, which are already five times the y-intercepts. This showed that all other values would be reliable. However, in this context, y-intercepts are not relevant, since at 0 µg/g of capsaicins and dihydrocapsaicins no peak area would be detected.

To check for the significant intercepts, we calculated percentage of y-intercept by dividing its value by the response of the 100% concentration response, multiplied by 100. We obtained values within ±2.0% both for capsaicin and dihydrocapsaicin, which are associated to the correlation coefficient which were more or equal to 0.999; we therefore considered that the standard curves are linear. For capsaicin, 100% concentration response was 9,392 (y-intercept 2.0%) and for dihydrocapsaicin it was 8,097 (y-intercept 1.8%). These limits are acceptable by the international guidelines for validation of the FDA.

Expected and actual concentration responses were plotted against expected concentrations (0.5, 0.25, 0.125 and 0.0625 µg/g) using four dilution factors (0, 2, 4 and 8). They both gave an equation (y = bx). The expected stock dilutions are more concentrated than the actual concentration dilutions which indicated that the dilutions are the concentrations expected (see Supplementary Data).

*Limit of Detection* (LOD) *and Limit of Quantitation* (LOQ) The method was validated by evaluating limit of detection (LOD) and limit of quantitation (LOQ) for both capsaicinoids. LOD and LOQ were estimated at an SD/b ratio of 3 and 10, where SD and b stand for the standard deviation of the slope and intercept of the regression line, respectively. The limit of detection (LOD) was 0.045 µg/kg and 0.151 µg/kg for capsaicin and dihydrocapsaicin, respectively. The limit of quantitation (LOQ) was 0.110 µg/kg and 0.368 µg/kg for capsaicin and dihydrocapsaicin, respectively.

*Reproducibility* An inter-day reproducibility (*n* = 30; acceptable by FDA and NATA) test was performed on capsaicin and dihydrocapsaicin for the UFLC method using four different chili pepper genotypes. The average relative standard deviations of the 30 replicate analysis of the inter-day reproducibility were represented in Table 2. This showed that the UFLC method is highly reproducible.

*Repeatability* An intra-day repeatability (*n* = 30) test was performed on capsaicin and dihydrocapsaicin for the UFLC method using four different chili pepper genotypes. The average relative standard deviations of the 30 replicate analysis of the intra-day repeatability were represented in Table 3. The result shows that the method is highly repeatable.

**Table 2:** Inter-day reproducibility data of the UFLC method for the determination of capsaicinoids in pepper (µg/kg).

	AVPP0705		AVPP0002		AVPP0805		C05573	
No. Sample	Cap	Dihy	Cap	Dihy	Cap	Dihy	Cap	Dihy
1	1908 ^(1)^	711	768	486	492	420	476	358
2	1868	690	811	485	502	433	466	360
3	1798	750	798	501	472	390	456	371
4	1867	701	779	499	501	387	500	350
5	1902	699	700	512	512	417	467	351
6	1998	680	801	501	511	401	480	354
7	1798	712	822	512	499	413	456	348
8	1811	718	783	493	518	429	489	359
9	1798	675	814	524	522	410	480	366
10	1928	700	780	505	498	386	457	379
11	1788	690	764	516	519	427	470	367
12	1901	721	817	507	510	388	469	346
13	1691	710	818	498	486	427	498	379
14	1800	724	808	509	532	430	481	380
15	1860	691	802	481	520	379	465	356
16	2198	736	821	521	472	424	488	345
17	1998	722	729	462	494	378	487	361
18	1878	716	813	481	514	415	497	344
19	1754	702	794	474	512	411	476	368
20	1791	683	765	535	508	402	486	376
21	1802	724	816	506	519	378	472	358
22	1855	731	807	527	481	367	459	364
23	1868	734	818	508	496	432	469	361
24	1801	745	799	487	477	421	480	357
25	1831	728	840	520	462	389	490	383
26	1808	767	788	491	530	398	487	377
27	1798	771	801	522	505	435	477	354
28	1818	747	810	502	528	426	485	362
29	1899	789	824	524	497	419	491	391
30	1861	724	775	515	474	399	475	365
Mean	1855.44	719.71	795.50	503.47	502.10	407.70	477.63	363.00
SD	92.37	27.50	29.31	17.29	18.76	19.45	12.53	11.99
RSD%	4.98	3.82	3.68	3.43	3.74	4.77	2.62	3.30

^(1)^ Values represent the mean of five replicate analysis; SD, standard deviation; RSD, relative standard deviation; Cap, capsaicin; Dihy, dihydrocapsaicin; *n* = 30.

*Precision and accuracy* Intra-day and inter-day precision data of the UFLC method were given in Table 4, indicating that the relative standard deviations are better than 5.0% for capsaicin and 9.9% for dihydrocapsaicin. Recovery experiments were performed using the standard addition method in order to study the accuracy of the UFLC method. The recovery of the added standard to the assay samples was calculated according to [26]:

Percentage recovery % = [(C_t_ − C_u_)/C_a_] × 100 (2)

where C_t_ is the total concentration of the analyte found, C_u_ is the concentration of the present analyte in the original AVPP0705, and C_a_ is the concentration of the pure analyte added to the original AVPP0705. The results were given in Table 4. The average recoveries obtained were quantitative (89.4%–90.1% for capsaicin, 92.4%–95.2% for dihydrocapsaicin), indicating good accuracy of the UFLC method.

**Table 3:** Intra-day repeatability data of the UFLC method for the determination of capsaicinoids inpepper (μg/kg).

	AVPP0705		AVPP0002		AVPP0805		C05573	
No. Sample	Cap	Dihy	Cap	Dihy	Cap	Dihy	Cap	Dihy
1	1778 ^(1)^	794	677	411	481	389	386	288
2	1801	789	687	401	488	367	381	298
3	1798	777	666	409	498	380	388	290
4	1890	698	657	418	468	381	387	295
5	1870	650	689	399	470	385	370	280
6	1786	699	670	389	484	379	377	279
7	1832	730	697	388	479	370	376	281
8	1799	786	678	390	480	377	369	286
9	1875	756	680	400	485	384	380	291
10	1800	790	681	412	489	386	383	278
11	1776	769	699	408	500	378	379	299
12	1854	798	657	403	496	390	385	296
13	1831	766	673	398	477	391	397	285
14	1876	801	674	405	465	394	390	294
15	1894	799	660	410	478	387	389	284
16	1799	800	664	409	473	369	375	287
17	1876	811	675	420	476	388	367	287
18	1865	737	674	419	483	376	378	284
19	1745	788	679	396	454	395	394	280
20	1789	776	672	386	495	385	392	283
21	1699	781	669	397	475	380	386	278
22	1855	787	681	408	476	382	374	289
23	1866	780	679	415	476	375	384	300
24	1886	769	665	414	497	367	382	276
25	1876	770	671	402	469	366	372	288
26	1856	779	677	412	472	387	378	277
27	1767	784	686	397	486	386	379	286
28	1803	793	676	386	465	388	383	281
29	1896	764	654	399	490	374	371	284
30	1876	813	649	407	477	385	369	273
Mean	1830.5	771.13	673.9	403.6	480.1	381.4	380.7	285.9
SD	50.76	36.13	11.76	9.83	11.03	8.11	7.87	7.16
RSD%	2.77	4.69	1.75	2.44	2.30	2.13	2.07	2.50

^(1)^ Values represent the mean of five replicate analysis; SD, standard deviation; RSD, relative standard deviation; Cap, capsaicin; Dihy, dihydrocapsaicin; *n* = 30.

**Table 4:** Precision and accuracy data of the UFLC for the determination of capsaicinoids in AVPP0705.

Component ^(1)^	Spiked amount (µg/kg)	Intra-day (%)	Inter day (%)	Recovery (%)
Capsaicin	1302	2.07	5.01	90.1
	3009	4.81	3.27	89.4
Dihydrocapsaicin	807.6	5.81	9.89	95.2
	3541	5.00	4.63	92.4

^(1)^ Sample weight approximately 3.0 g; Concentration of capsaicin and dihydrocapsaicin in the initial sample was 13,076 and 7,155 µg/kg, respectively; *n* = 3.

### 2.3. Analysis of Capsaicinoids in Samples

**Figure 3:**
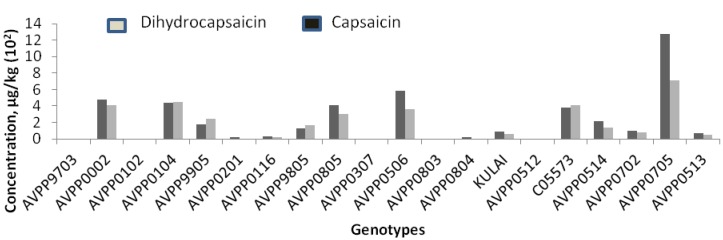
Capsaicin and dihydrocapsaicin obtained using acetonitrile as extraction solvent.

The high-speed analysis of the UFLC method was considered as providing good-efficiency analysis and to be environmentally friendly. The UFLC method was applied to determine the content of capsaicin and dihydrocapsaicin contents of twenty-one pepper genotypes and their corresponding pungency levels. The chromatograms attached (supplementary data) correspond to an extracted solution of some genotypes. From the chromatograms obtained from the studied chili peppers, the main peaks of interest identified among the capsaicinoids were capsaicin and dihydrocapsaicin. The UV absorption spectra corresponding to capsaicin and dihydrocapsaicin peaks were obtained from the photo diode array detector (PDA). The ultraviolet detection wavelength was set at 280 nm for all the capsaicinoids, because it is the maximum absorbance for both capsaicinoids. The chromatogram showed a complete separation between the two elements (capsaicin and dihydrocapsaicin) and no interference with other capsaicinoid peaks. The capsaicinoid contents are calculated and presented in Figure 3. The amount of capsaicin and dihydrocapsaicin from the chili pepper samples were found to differ significantly (*p* > 0.05). It ranged from 0–13,076 µg/kg and 0–7,155 µg/kg for both capsaicin and dihydrocapsaicin, respectively, as shown in Table 5. Genotype AVPP0705 was found to record the highest capsaicin content and was the highest in pungency which was significantly (*p* > 0.05) higher than all the other samples tested. Genotypes AVPP9703, AVPP0512, AVPP0307, AVPP0803 and AVPP0102 were found to record no capsaicin and therefore be non-pungent. Similar variation in capsaicin content of different peppers has been previously reported [27,28,29].

**Table 5:** The capsaicinoids content of the twenty-one chilli pepper genotype samples (µg/kg).

Genotypes	Capsaicin	Dihydrocapsaicin	Total Capsaicinoids
AVPP0705	13076	7155	20231
AVPP0506	5945	2999	8944
AVPP0104	4283	4698	8981
AVPP0002	4945	4346	9291
C05573	2989	4280	7269
AVPP0805	4230	3340	7570
AVPP9905	2054	2218	4272
AVPP0904	2012	1613	3625
AVPP0514	2468	1470	3938
AVPP9805	1248	1568	2816
AVPP0702	1524	850	2374
KULAI	799	606	1405
AVPP0513	892	553	1445
AVPP0116	299	246	545
AVPP0804	191	ND	191
AVPP0201	186	ND	186
AVPP9703	ND	ND	ND
AVPP0512	ND	ND	ND
AVPP0307	ND	ND	ND
AVPP0803	ND	ND	ND
AVPP0102	ND	ND	ND

*n* = 2.

For all the chili pepper samples, the correlation between Scoville heat unit (SHU) and the two capsaicinoids obtained was calculated as shown in Table 5 and Table 6 by using the relationship between this content (µg/kg) and its SHU rating of approximately 15 SHU equivalents to 10 µg/kg of capsaicinoids [30]. Therefore, their corresponding SHU were found in the range of 0-237,245 SHU. From these results, it is indicated that capsaicin and dihydrocapsaicin were primarily responsible for the SHU rating. Thus, the chili sample AVPP0705 gave quite a high SHU related with its higher content of the capsaicinoids. Therefore, total yields of capsaicinoids in these chili peppers ranged from 0–20,231 µg/kg. In addition, capsaicin and dihydrocapsaicin have the same trend in contents of the capsaicinoids, and in particular capsaicin was found in higher contents than dihydrocapsaicin in all samples studied except C05573, AVPP9905 and AVPP9805. Genotypes were classified into five different classes viz: very highly pungent, highly pungent, moderately pungent, mildly pungent and non-pungent as shown in Table 6. AVPP0705 recorded the highest while AVPP9703, AVPP0512, AVPP0307, AVPP0803 and AVPP0102 were recorded as non-pungent.

### 2.4. Percentage Capsaicin Content

The number of SHUs of the pepper in isolation is not the critical factor. The most important factor is the capsaicin content. All peppers used in this study, fall outside the pungency limit (0.5%–0.9%) presented by the BPC (British Pharmaceutical Codex) [31] except AVPP0506, AVPP0104, AVPP0002, C05573 and AVPP0805 that fall within the pungency limit (Table 6), hence could be recommended for oleoresin production, which is used in the formulation of certain pharmaceuticals. Despite the fact that AVPP0705 gave the highest capsaicin content, it would not be recommended for pharmaceutical Industry because the percentage capsaicin content is high (1.5%) as there have been no proof it is safe for human use [32,33]. Therefore, on the basis of capsaicin content, only AVPP0506, AVPP0104, AVPP0002, C05573 and AVPP0805 can serve as potential sources of capsaicin for use in the pharmaceutical industry.

**Table 6:** The % capsaicin content, Scoville heat units, and degree of pungency of twenty-one chilli pepper genotype samples (dry weight).

Genotypes	% Capsaicin Content	Scoville Heat Unit	Degree of Pungency
AVPP0705	1.49	237245	very highly pungent
AVPP0506	0.66	104888	very highly pungent
AVPP0104	0.69	110796	very highly pungent
AVPP0002	0.65	104678	very highly pungent
C05573	0.57	91097	very highly pungent
AVPP0805	0.56	88906	very highly pungent
AVPP9905	0.30	47946	highly pungent
AVPP0904	0.30	47372	highly pungent
AVPP0514	0.28	44259	highly pungent
AVPP9805	0.22	35769	highly pungent
AVPP0702	0.14	22146	moderately pungent
KULAI	0.13	20564	moderately pungent
AVPP0513	0.13	20566	moderately pungent
AVPP0116	0.04	7170	moderately pungent
AVPP0804	0.02	2767	mildly pungent
AVPP0201	0.02	3020	mildly pungent
AVPP9703	0	0	non-pungent
AVPP0512	0	0	non-pungent
AVPP0307	0	0	non-pungent
AVPP0803	0	0	non-pungent
AVPP0102	0	0	non-pungent

*n* = 2.

## 3. Experimental

### 3.1. Instrument and Apparatus

Ultra-fast liquid chromatography was carried out using a Shimadzu Ultra XR (LC- 20AD × R) system (Columbia, SC, USA) equipped with a SPD-M20A prominence Diode Array detector, SK- 20A × R auto sampler and CTO- 20A column oven. Detection was conducting using a UV absorption detector. Identification of capsaicinoids was achieved through comparison of retention times of each capsaicinoids of the chilli samples with those of standard compounds.

### 3.2. UFLC Analytical Conditions

Column: Purospher^®^ STAR RP-18 e (150 mm × 4.6 mm × 5 µm)
Mobile phase: 1.0% Acetic Acid aq./Acetonitrile = 1/1 (v/v)
Flow rate: 1.2 mL
Column Temp: 30 °C
Detection: SPD-M20 A at 280 nm
Injection Vol.: 2 µL
Data acquisition time: Sampling = 6.25 Hz; Time constant = 0.160 s

### 3.3. Samples

Twenty one genotypes of chili pepper seeds were collected from AVRDC, Taiwan, and grown under heat condition (Table 7). Whole ripe fruits were harvested and dried for capsaicinoid extraction and analysis. The extraction of capsaicin from the chili pepper samples was done using the method described by [1] and capsaicinoids levels were analyzed using ultra fast liquid chromatography. A sample for assay consisted of 5–8 fruits depending on the size of the chili fruit. The extraction and quantitation was carried out in duplicate for each genotype. The fruits were oven-dried at 60 °C 2–5 days (depending on the fruit size), grounded using laboratory mill. The grounded samples were stored in sealed plastic bags at 20 °C until processed.

**Table 7:** Genotypes and their degree of heat tolerance.

Genotypes	Degree of tolerance *
AVPP0705	Tolerant
AVPP0506	Tolerant
AVPP0104	Moderately Tolerant
AVPP0002	Sensitive
C05573	Tolerant
AVPP0805	Tolerant
AVPP9905	Tolerant
AVPP0904	Tolerant
AVPP0514	Tolerant
AVPP9805	Tolerant
AVPP0702	Tolerant
KULAI	Moderately Tolerant
AVPP0513	Tolerant
AVPP0116	Tolerant
AVPP0804	Tolerant
AVPP0201	Tolerant
AVPP9703	Sensitive
AVPP0512	Tolerant
AVPP0307	Tolerant
AVPP0803	Tolerant
AVPP0102	Moderately Tolerant

* Data not shown.

### 3.4. Reagents

Analytical grade acetonitrile (99.9%) and methanol (100%) were purchased from Sigma-Aldrich (St. Louis, MO, USA). Glacial acetic acid (99.8%) was from R & M Marketing (Essex, UK). Capsaicin (>95%) and dihydrocapsaicin (~90%) were purchased from Sigma-Aldrich (St. Louis, MO, USA). Stock solution of each capsaicinoids to be determined was prepared by weighing accurately 50 mg and dissolving each compound in 100% methanol. These solutions were stored at 4 °C and used for the preparation of diluted standard solution in methanol.

### 3.5. Extraction of Capsaicinoids

For the capsaicinoid extraction, a 1:10 (g/mL) ratio of dried chili powder to acetonitrile was placed in 120 mL glass bottles. Bottles were capped and placed in an 80 °C water bath for 4 h; they were swirled manually every hour. Samples were removed from the water bath and cooled to room temperature. Two to 3 mL of supernatant was extracted and filtered (0.45 µm filter on a 5-mL disposable syringe) into a 2-mL glass sample vial, capped and stored at 4 °C until analyses [1]. A 2 µL aliquot was used for each UFLC injection. For each variety, extraction and analysis was carried out in duplicate.

### 3.6. Conversion to Scoville Heat Units *(SHU)*

Scoville Heat Units was used to calculate the heat for all samples. Scoville Heat Units are calculated in parts per million of heat (ppmH) based on sample dry weight according to the following formula [32]:

ppmH = [Peak area of capsaicin + (0.82) (peak area of dihydrocapsaicin)] (ppm standard) (mL acetonitrile)/(Total capsaicin peak area of standard) (g sample)
(3)

Conversion to Scoville Heat Units was made by multiplying ppmH by a factor of 15.

### 3.7. Percentage Capsaicin Content

The determination of capsaicin content was performed according to the method described by [33]:

A divided by B times the percentage of pepper = capsaicin Content
(4)

where A = Scoville Heat Units claimed, B = 16 Million SHUs which is the rating for 100% pure capsaicin and % Pepper = percentage of pepper claimed

### 3.8. Statistical Analysis

ANOVA for the capsaicin, dihydrocapsaicin, and total capsaicinoid content data for the genotypes was carried out according to the general linear model (GLM), using the SAS software package *v*ersion 9.2 (SAS Institute Inc., Cary, NC, USA). Means were compared using the LSD test. To estimate the suitability of the qualitative analysis to distinguish degrees of pungency, ANOVA of capsaicin, dihydrocapsaicin, and total capsaicinoid content data for the qualitative categories was carried out. Means were compared using Duncan’s and LSD test.

## 4. Conclusions

The results from this experiment showed that the UFLC method can be applicable to the chili pepper variety, demonstrating excellent separation without hindrance of any interference. AVPP0705, AVPP0506, AVPP0104, AVPP0002, C05573 and AVPP0805 are the most pungent genotypes among the peppers studied. A few genotypes AVPP9703, AVPP0512, AVPP0307, AVPP0803 and AVPP0102 that recorded 0 SHU (non-detect) where found to be non-pungent. Others fall between moderately and mildly pungent genotypes. This shows that, with exception of AVPP9703, AVPP0512, AVPP0307, AVPP0803 and AVPP0102, all pepper genotypes studied can serve as potential sources of capsaicin. On the other hand, only genotypes AVPP0506, AVPP0104, AVPP0002, C05573 and AVPP0805 would be recommended as potential source of capsaicin for the pharmaceutical industries.

## Supplementary Materials

Supplementary materials can be accessed at: http://www.mdpi.com/1420-3049/19/5/6474/s1.

## References

[1] Collins M.D., Mayer-Wasmund L., Bosland P.W. (1995). Improved method for quantifying capsaicinoids in C*apsicum* using high performance liquid chromatography. HortScience.

[2] Peña-Alvarez A., Ramírez-Maya E., Alvarado-Suárez L.A. (2009). Analysis of capsaicin and dihydrocapsaicin in peppers and pepper sauces by solid phase microextraction–gas chromatography–mass spectrometry. J. Chromatogr. A.

[3] Caterina M.J., Schumacher M.A., Tominaga M., Rosen T.A., Levine J.D., Julius D. (1997). The capsaicin receptor: A heatactivated ion channel in the pain pathway. Nature.

[4] Caterina M.J., Leffler A., Malmberg A.B., Marti W.J., Trafton J., Petersen-Zeitz K.R., Koltzenburg M., asbaum A.I., Julius D. (2000). Impaired nociception and pain sensation in mice lacking the capsaicin receptor. Science.

[5] Chu C.J., Huang S.M., de Petrocellis L., Bisogno T., Ewing S.A., Miller J.D., Zipkin R.E., Daddario N., Appendino G., Di Marzo V. (2003). *N*-Oleoyldopamine, a novel endogenous capsaicin-like lipid that produces hyperalgesia. J. Biol. Chem..

[6] Lee R.J., Yolton R.L., Yolton D.P., Schnider C., Janin M.L. (1996). Personal defense sprays: Effects and management of exposure. J. Am. Optom. Assoc..

[7] Iwai K., Suzuki T., Fujiwake H. (1979). Formation and accumulation of pungent principle of hot pepper fruits, capsaicin, and its analogues, in *Capsicum annuum* var. *annuum* cv. Karayatsubusa at different stages of flowering. Agric. Biol. Chem..

[8] Iida T., Moriyama T., Kobata K. (2003). TRPV1 activation and induction of nociceptive response by a non-pungent capsaicin-like compound, capsiate. Neuropharmacology.

[9] Backonja M.M., Malan T.P., Vanhove G.F., Tobias J.K. (2010). NGX-4010, a high-concentration capsaicin patch, for the treatment of postherpetic neuralgia: A randomized, double-blind, controlled study with an open-label extension. Pain Med..

[10] Tesfaye S. (2009). Advances in the management of diabetic peripheral neuropathy. Curr. Opin. Support. Palliat. Care.

[11] Derry S., Lloyd R., Moore R.A., McQuay H.J. (2009). Topical capsaicin for chronic neuropathic pain in adults. Cochrane Database Syst. Rev..

[12] Reyes-Escogido M.L., Gonzalez-Mondragon E.G., Vazquez-Tzompantzi E. (2011). Chemical and Pharmacological Aspects of Capsaicin. Molecules.

[13] DeWitt D., Bosland P.W. (1993). The Pepper Garden.

[14] Contreras-Padilla M., Yahia E.M. (1998). Changes in capsaicinoids during development, maturation, and senescence of chili peppers and relation with peroxidase activity. J. Agric. Food Chem..

[15] Estrada B., Bernal M.A., Diaz J., Pomar F., Merino F. (2000). Fruit development in *Capsicum annuum*: Changes in capsaicin, lignin, free phenolics, and peroxidase patterns. J. Agric. Food Chem..

[16] Estrada B., Bernal M.A., Diaz J., Pomar F., Merino F. (2002). Capsaicinoids in vegetative organs of *Capsicum annuum* L. in relation to fruiting. J. Agric. Food Chem..

[17] Harvell K., Bosland P.W. (1997). The environment produces a significant effect on the pungency of chilis. HortScience.

[18] Estrada B., Diaz J., Merino F., Bernal M.A. (1999). The effect of seasonal changes on the pungency level of Padron pepper fruits. Capsicum Eggplant Newsl..

[19] Garces-Claver A., Arnedo-Andre’s M.S., Abadia J., Gil-Ortega R., Alvarez-Fernandez A. (2006). Determination of capsaicin and dihydrocapsaicin in *Capsicum* fruits by liquid chromatographyelectrospray/time-of-flight mass spectrometry. J. Agric. Food Chem..

[20] Reilly C.A., Crouch D.J., Yost G.S., Fatah A.A. (2002). Determination of capsaicin, nonivamide, and dihydrocapsaicin in blood and tissue by liquid chromatography-tandem mass spectrometry. J. Anal. Toxicol..

[21] Thompson R.Q., Phinney K.W., Welch M.J., White V.E. (2005). Quantitative determination of capsaicinoids by liquid chromatography-electrospray mass spectrometry. Anal. Bioanal. Chem..

[22] Schweiggert U., Carle R., Schieber A.  (2006). Characterization of major and minor capsaicinoids and related compounds in chili pods (*Capsicum frutescens* L.) by high-performance liquid chromatography-atmospheric pressure chemical ionization mass spectrometry. Anal. Chim. Acta.

[23] Scoville W.L. (1912). Note on *Capsicum*. J. Am. Pharm. Assoc..

[24] Weiss E.A. (2002). Spice Crops.

[25] George W.L. Official Methods of Analysis of AOAC International. http://www.aoac.org/iMIS15_Prod/AOAC/Publications/Official_Methods_of_Analysis.

[26] Ha J., Seo H.Y., Shim Y.S., Seo D.W., Seog H., Ito M., Nakagawa H. (2010). Determination of capsaicinoids in foods using ultra high performance liquid chromatography. Food Sci. Biotechnol..

[27] Nwokem C.O., Agbaji E.B., Kagbu J.A., Ekanem E.J. (2010). Determination of capsaicin content and pungency level of five different peppers grown in Nigeria. NY Sci. J..

[28] Othman Z.A.A., Ahmed Y.B.H., Habila M.A., Ghafar A.A. (2011). Determination of capsaicin and Dihydrocapsaicin in *Capsicum* Fruit samples using High Performance Liquid Chromatography. Molecules.

[29] Sanatombi K., Sharma G.J. (2008). Capsaicin content and pungency of different *Capsicum* spp. cultivars. Not. Bot. Horti. Agrobot. Cluj. Napoca..

[30] Mathur R., Dangi R.S., Das S.C., Malhotra R.C. (2000). The hottest chilli variety inIndia. Curr. Sci. India.

[31] Pharmaceutical Society of Great Britain (1973). British Pharmaceutical Codex.

[32] American Spice Trade Association (2004). Official Analytical Methods of the American Spice Trade Association.

[33] Pepper Enforcement. http://www.pepperenforcement.com/capsaicin.html.

