# Supplementary material for: Capsaicin and Dihydrocapsaicin Determination in Chili Pepper Genotypes Using Ultra-Fast Liquid Chromatography
Source: Molecules. 2014 May 21;19(5):6474–88. doi: 10.3390/molecules19056474 (PMC6271280; doi:10.3390/molecules19056474)
Supplement: Supplementary file 1 [file molecules-19-06474-s001.pdf]

Supplementary File

Figure S1. Calibration curve for Capsaicin.

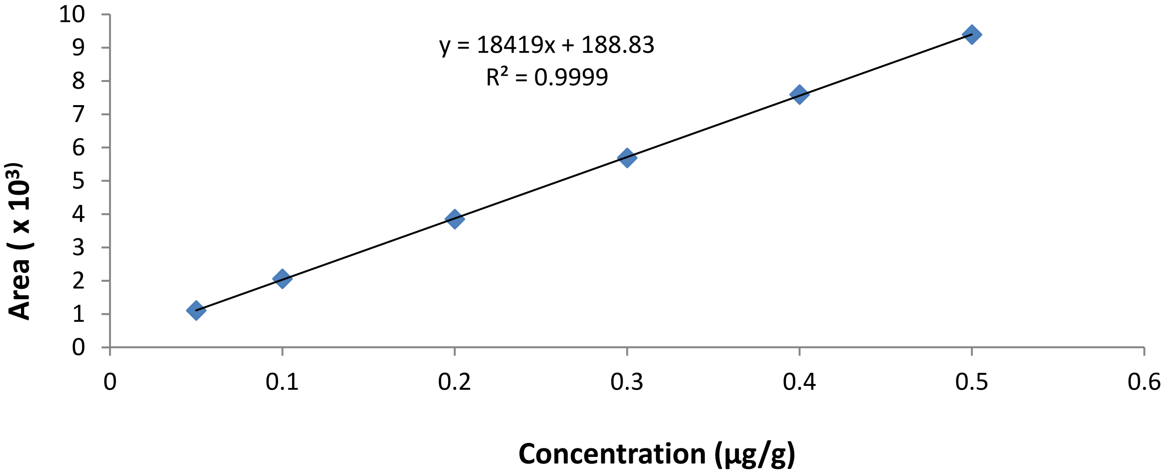

Figure S2. Calibration curve for Dihydrocapsaicin.

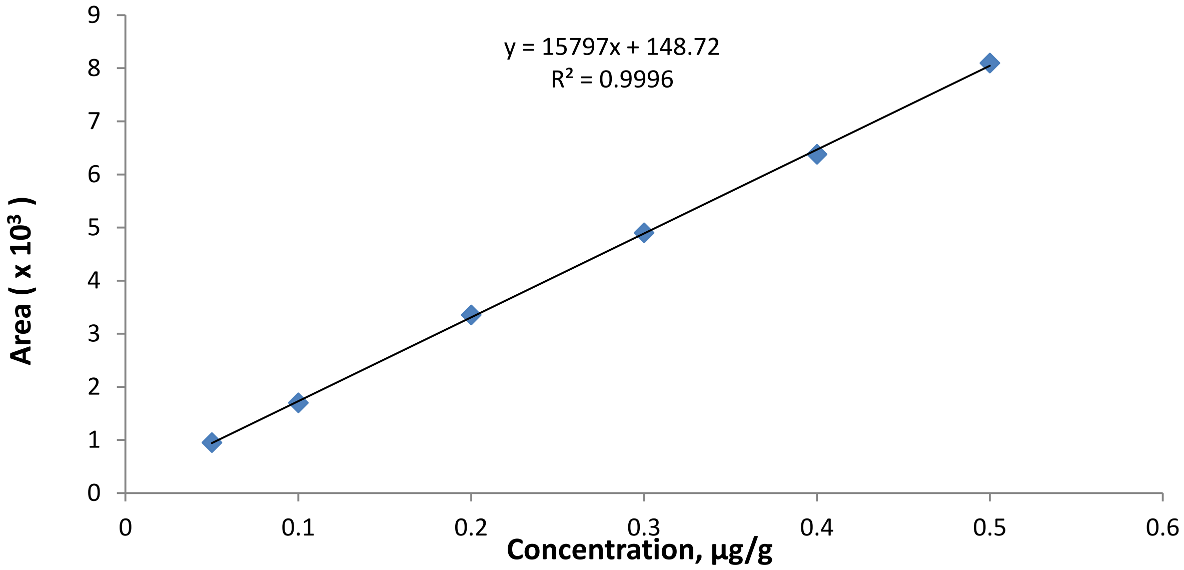

Figure S3. Expected and actual response plotted against expected concentrations for capsaicin.

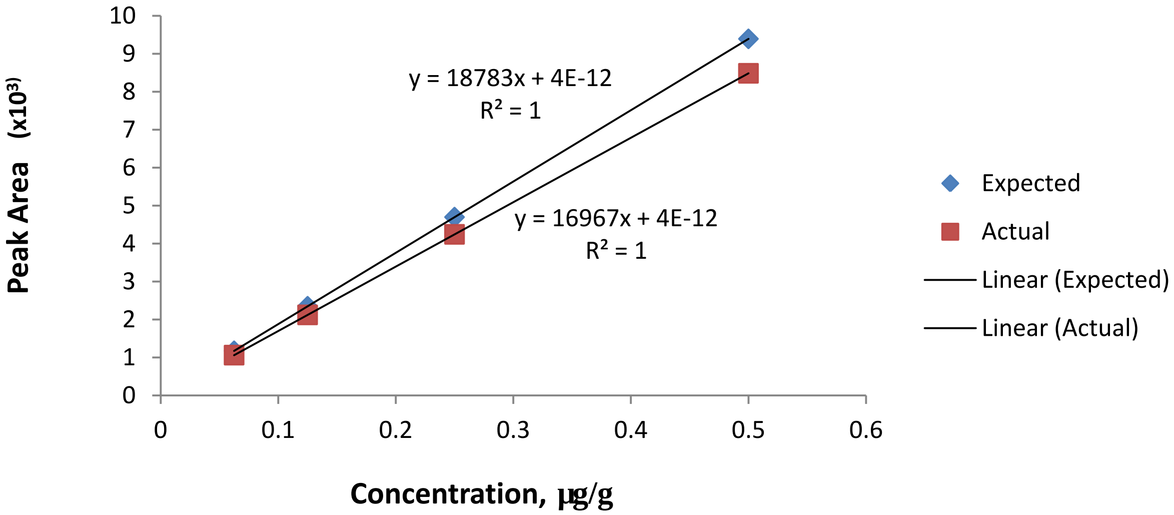

Figure S4. Expected and actual response plotted against expected concentrations for dihydrocapsaicin.

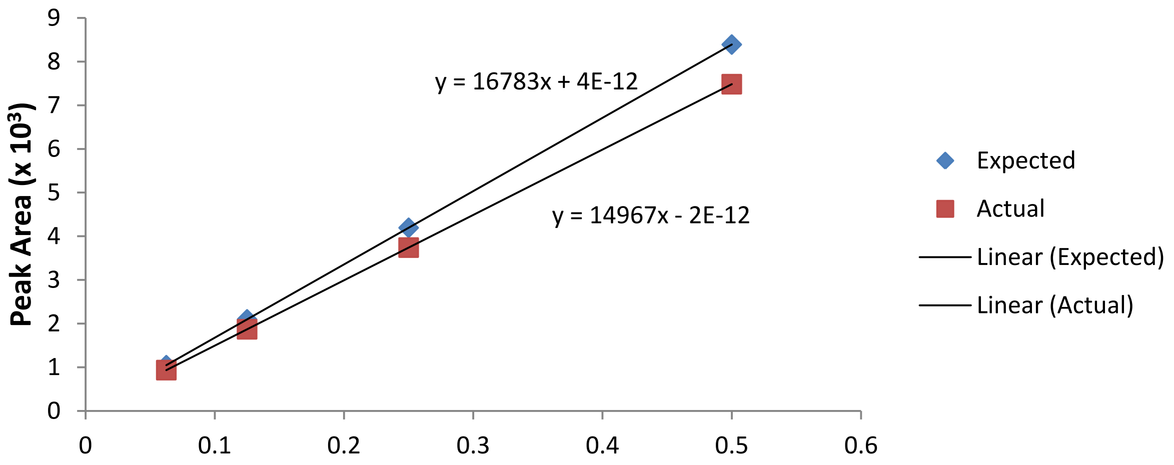

Figure S5. The chromatogram of an extracted solution of some genotypes. (**a**) Blank Sample (AVPP0803) – Non pungent; (**b**) AVPP0514 (Highly Pungent); and (**c**) C05573 (Very Highly Pungent).

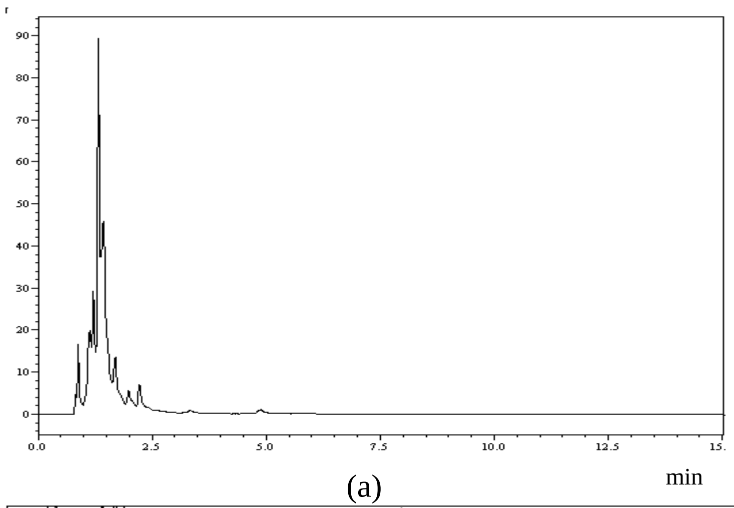

(a)

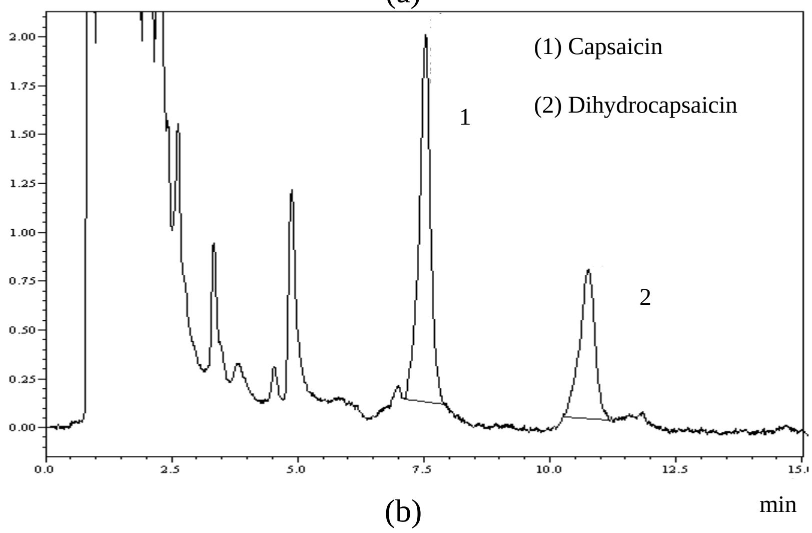

(b)

Figure S5. *Cont.*

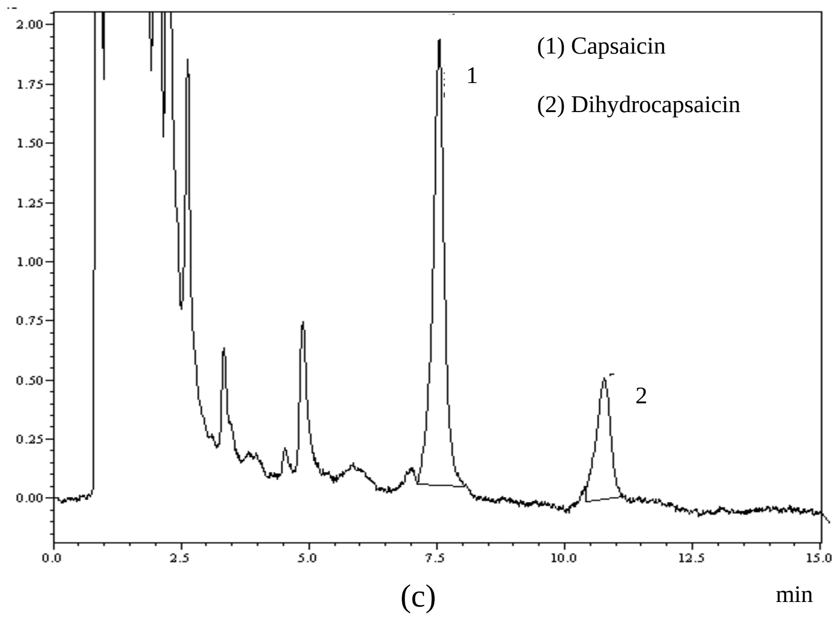

(c)
